# The Regulation of Autophagy by Influenza A Virus

**DOI:** 10.1155/2014/498083

**Published:** 2014-03-23

**Authors:** Rong Zhang, Xiaojuan Chi, Song Wang, Baomin Qi, Xiaoqiang Yu, Ji-Long Chen

**Affiliations:** ^1^College of Animal Science, Fujian Agriculture and Forestry University, Fuzhou 350002, China; ^2^CAS Key Laboratory of Pathogenic Microbiology and Immunology, Institute of Microbiology, Chinese Academy of Sciences (CAS), Beijing 100101, China; ^3^Division of Cell Biology and Biophysics, School of Biological Sciences, University of Missouri, Kansas City, MO 64110, USA

## Abstract

Influenza A virus is a dreadful pathogen of animals and humans, causing widespread infection and severe morbidity and mortality. It is essential to characterize the influenza A virus-host interaction and develop efficient counter measures against the viral infection. Autophagy is known as a catabolic process for the recycling of the cytoplasmic macromolecules. Recently, it has been shown that autophagy is a critical mechanism underlying the interaction between influenza A virus and its host. Autophagy can be induced by the infection with influenza A virus, which is considered as a necessary process for the viral proliferation, including the accumulation of viral elements during the replication of influenza A virus. On the other hand, influenza A virus can inhibit the autophagic formation via interaction with the autophagy-related genes (Atg) and signaling pathways. In addition, autophagy is involved in the influenza virus-regulated cell deaths, leading to significant changes in host apoptosis. Interestingly, the high pathogenic strains of influenza A virus, such as H5N1, stimulate autophagic cell death and appear to interplay with the autophagy in distinct ways as compared with low pathogenic strains. This review discusses the regulation of autophagy, an influenza A virus driven process.

## 1. Introduction

Influenza A virus causes significant morbidity and mortality and enormous economic losses annually in the world. It is an enveloped negative-sense RNA virus and its genome possesses eight segments encoding 13 proteins [1]. Some of the eight viral RNAs (vRNAs) have been shown to encode more than one polypeptide [2]. Recently, another novel protein, PA-X, from influenza A virus has been discovered as a product of ribosomal frameshifting [3]. Part of PA-X is encoded by a specific open reading frame of PA. Additionally it has been reported that PA-X is associated with the host response and viral virulence [2, 3]. Hemagglutinin (HA) and neuraminidase (NA) are both surface antigenic glycoproteins of influenza A virus that play key roles in the viral pathogenesis [4, 5]. On the inside surface of bilayer lipid membranes there are M1 and M2 (one- and two-matrix protein) [6, 7]. Eight segments of viral genome RNA are contained in the envelope in the form of helical ribonucleoprotein. One segment encoded the nucleocapsid (NP) protein associated with packing and transport of viral RNA. NP together with the small amount of proteins, PB1, PB2, PA, is involved in the activity of RNA-dependent RNA polymerase. The nonstructural (NS1) protein coded by the smallest segment of vRNA is a multifunctional protein in viral infection. NS1 plays an important role in restricting host innate immune response via the inhibition of the type I interferon (IFN) as well as the dsRNA-dependent protein kinase R (PKR) [8, 9]. Recently, the highly pathogenic influenza A virus has resulted in enormous economic losses due to its morbidity and mortality. Moreover, outbreak of influenza epidemics and pandemics has become one of the most serious threats to the human population. Therefore, it is necessary to characterize additional mechanisms of virus-host interaction for developing efficient countermeasures against the viral infection.

Autophagy is just one of such mechanisms involved in influenza A virus infection. Thus, more and more attention has been paid to the complicated relationship between the virus infection and autophagy machinery. Although the autophagy pathway is recognized as a component of host defense, growing evidence suggested that autophagy machinery has been utilized by certain viruses, including influenza virus [10]. Autophagy, or “self-eating”, and the ubiquitin-proteasome degradation pathway are the two essential catabolic processes for eliminating superfluous and harmful proteins in eukaryotic cells [10, 11]. Importantly, autophagy can target the protein aggregates and entire organelles that are too large for proteasome to degrade [11]. In this way, autophagy drives a cytoplasmic material cycle for cell survival under the harsh condition such as starvation [12]. Moreover, autophagy also removes the pathogenic protein aggregates from the cytoplasm, preventing viral infection [10]. Therefore, autophagy plays an important role in regulating homeostasis in cellular stress response [13]. During autophagy, an isolation membrane, or “phagophore,” forms in the cytoplasm and expands to engulf the targeting portion of cytoplasm, leading to the formation of a double-membrane vesicle termed autophagosome. This autophagosome can fuse with late endosome and eventually fuse its outer membrane with the lysosome, resulting in the formation of autolysosomes with concomitant degradation of the inner autophagosome membrane and the autophagosomal contents by lysosomal hydrolysis [10, 13]. The molecular mechanism of the autophagy membrane dynamics is initially characterized by the autophagy-related genes (Atgs), and more than 30 Atgs have been discovered in yeast [14]. However, the processes are so complex that functions of Atgs are still not fully understood. Furthermore, the identification of Atg homologs in higher eukaryotes could better understand the molecular mechanism of autophagy in eukaryote cells. The elongation and closure of the isolation membrane are relevant to two ubiquitin-like systems: the Atg12-Atg5-Atg16L1 and Atg8-PE (the microtubule-associated protein 1 light chain 3 (LC3) in mammalian cells), or LC3-II, protein conjugation pathway. In the Atg12-Atg5-Atg16L1 system, activated Atg12 conjugated to Atg5, which then, linked and interplayed with a coiled-coil protein Atg16L1. This protein could attach the Atg12-Atg5-Atg16L1 complex to the outer membrane of phagophore. This complex works as mechanical stabilization and might be involved in the curvature of the forming autophagosome [12, 15]. In the second system, LC3, a soluble protein, is initially distributed in the cytosol. After being cleaved by the cysteine protease Atg4, it becomes LC3-I, which is subsequently conjugated to phosphatidylethanolamine on the surface of autophagosome membrane. This LC3-phosphatidylethanolamine conjugation is termed LC3-II, which is required for closure of isolation membrane and autophagosome fusion with lysosome [12].

The better understanding of pathogenesis caused by influenza A virus critically requires the analysis of autophagic mechanism in infected cells and the regulation of autophagy by influenza A virus. The parasitic characteristic of influenza viruses prompts them to evade the host cell defense and utilize cellular sources to accelerate their proliferation. However, molecular basis of these processes remains elusive.

## 2. Infection of Influenza A Virus Promotes the Formation of Autophagosome

It is known that the autophagosome is an essential intermediate structure during autophagy. Thus, presence of autophagosomes in the cytoplasm indicates the induction of autophagy. Growing evidence suggests that infection by influenza A virus increases the generation of autophagosomes, which could be detected by electron microscopy, green fluorescent protein (GFP) labeled LC3, and biochemical analysis of LC3 [16].

### 2.1. Influenza A Virus Induces Autophagosome and Increases the Levels of LC3-II

The mammalian target of rapamycin TOR (mTOR) kinase is one of the most important inhibitory factors for autophagy. Thus, inactivation of mTOR leads to autophagy stimulation. Rapamycin, which represses the mTOR activation, can be used to induce autophagy. Strikingly, it has been shown that the formation of autophagosomes greatly increased in the rapamycin-treated, H1N1 or H9N2 influenza A virus-infected Madin-Darby canine kidney (MDCK) cells compared with the mock-infected cells [17]. Although LC3 is located throughout cytoplasm, it is redistributed after being recruited by the autophagic vesicles. Therefore, LC3 serves as a marker of the autophagosome formation. In particular, LC3-II stays with autophagosomes during autophagy, until the autophagosome fusion with lysosomes. Upon this fusion, LC3-II is degraded together with autophagosome cargo and the inner isolation membrane by lysosomal enzymes. Thus, the LC3-II level can be used to monitor autophagy process [15, 18]. The observation using fluorescence microscope showed that characteristic punctate GFP-LC3 proteins accumulated both in the cells treated with rapamycin and in the cells infected with the influenza A virus, indicating that the autophagosomes were induced in both rapamycin-treated and influenza A virus-infected cells. Furthermore, the accumulation of membrane vesicles, which contain enveloped organelles and cellular contents, was identified in the infected cells by the electron microscopists [16, 17]. Additionally, in the influenza virus-infected cells, there are numerous small autophagosomes that are distributed with high mobility and one large immobile vesicle is localized at the perinuclear space. In contrast, in uninfected cells, few autophagosomes were observed in the cytoplasm [6]. Consistent with the electron microscopy observations, the biochemical assays of LC3 identified the induction of autophagosomes. Through the inhibition of lysosomal proteases, an increased level of LC3-II observed at 6 hours and 12 hours after infection further confirmed that autophagy is stimulated in the influenza-infected cells at early stages of infection [19].

### 2.2. M2, HA, and NS1 Proteins of Influenza A Virus Are Involved in the Induction of Autophagy

**Figure 1:**
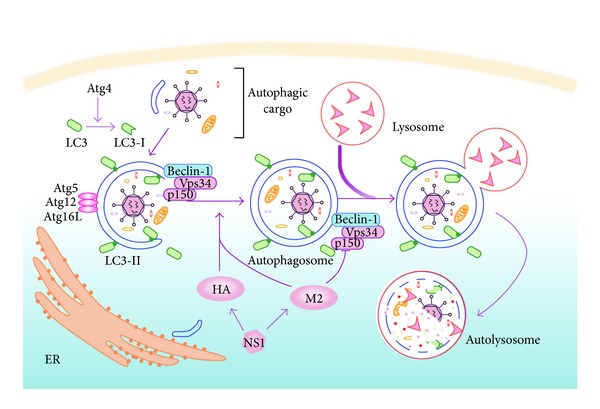
Autophagy machinery in infected cell. An isolation membrane initially forms around the targeting viruses or macromolecules. It is considered that this isolation membrane originates from the endoplasmic reticulum (ER), and its formation is modulated by PI3K complex which is comprised of Vps34, p150, and Beclin-1. The elongation and closure of the autophagosomes are relevant to two ubiquitin-like systems: the Atg12-Atg5-Atg16L1 and LC3-II protein conjugations. After enwrapping the autophagic cargos, the autophagosome fuses with the lysosome, resulting in the acidification of autolysosome which is concomitant with degradation of the inner autophagosome membrane and the autophagosomal contents by lysosomal hydrolysis. The viral proteins of influenza A virus induce both positive and negative regulation of the autophagy pathway.

Previous studies showed that only live Influenza A virus can trigger the accumulation of autophagosomes, indicating that it is the viral infection and replication that are essential to induce autophagy. In recent studies, M2 is emerging as a critical protein of influenza A virus to regulate autophagy in host. M2 alone is sufficient to induce the initial steps of autophagosome formation [6, 17, 20], whereas other proteins of influenza A virus, including PB1, PA, PB2, NP, NEP, NS1, M1, and NA, might not stimulate autophagy alone. In addition, viral HA protein could slightly activate autophagy. For example, it was found that the cleavage products of H5 and H7 caused a notable upregulation of LC3-II protein [20]. The subtype H5 from H5N1 virus might also be related to the stimulation of autophagic cell death [21]. The NS1, a known multifunction protein of influenza A virus, has been reported to inhibit apoptosis at the early stage of infection for viral replication [22]. Interestingly, recent studies suggest that NS1 could upregulate the autophagy [20, 23]. Even though seldom provoking the autophagy alone, the NS1 contributes to increasing the synthesis of HA and M2 and thereby induces the autophagy indirectly (Figure 1).

### 2.3. High Pathogenic Influenza A Virus Induces Autophagy by Regulating the Akt-TSC2-mTOR Signaling Pathway

**Figure 2:**
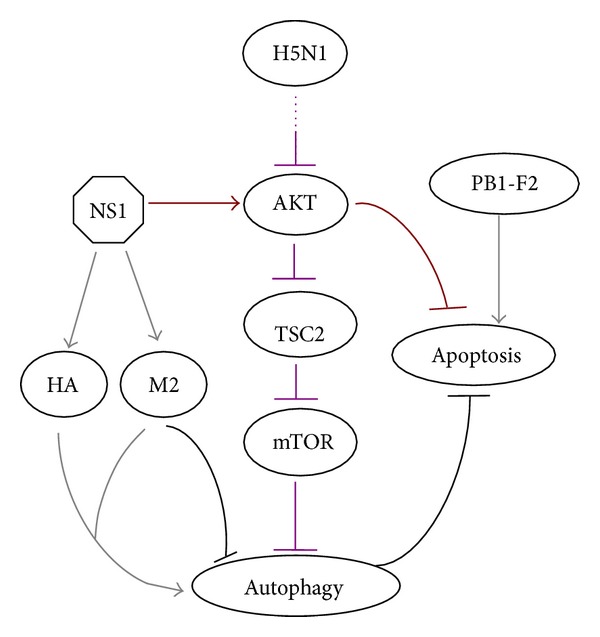
The regulatory pathways of autophagy by influenza A virus.

The H5N1 strain is a highly pathogenic Influenza A virus rendering significant mortality, which may result from the virus-induced autophagic cell deaths. It has been shown that H5N1 induces the autophagy via the inhibition of mTOR, which interplays with Atg1 (ULK1 in the mammalian) and triggers ULK1 as well as mammalian Atg13 to suppress autophagy [24–27]. On the other hand, the tumor suppressor protein 2 (TSC2) is an upstream inhibitor of mTOR. The TSC2-deficient model demonstrates that the silencing of TSC2 by RNA interference markedly inhibits the H5N1-induced autophagy and reduces the A549 cell death. Therefore, H5N1 stimulates the mTOR-related autophagy pathway likely through regulating the TSC2 expression [21, 28, 29]. Further results reveal that influenza A virus H5N1 notably decreases the phosphorylation of the AKT kinase that downregulates the TSC2. Collectively, H5N1 may induce the autophagy by modulating the Akt-TSC2-mTOR pathway (Figure 2). However, further studies are needed to better understand the precise regulatory mechanism [21, 28].

## 3. Influenza A Virus Inhibits the Degradation of Autophagosome

Autophagosomes are known as transient vesicles which are soon degraded by lysosomes. Thus, the accumulation of influenza A virus-induced autophagosomes could represent either increased formation or reduced degradation of autophagosomes [30]. Recent reports show that influenza A virus not only triggers the initial steps of autophagosome formation but also prevents the final steps of autophagosome maturation. By the fluorescence microscopy, both the labeled acidified lysosomes and the stained lysosome-associated membrane protein 1 (LAMP1) did not colocalize with GFP-LC3 in the influenza-infected cells, indicating that autophagosomes maturation process was blocked [6]. To further verify this consequence, an mRFP-GFP-LC3 tandem construct was used to trace autophagosomes and autolysosomes. When the mRFP-GFP-LC3 was delivered into acidified lysosomes, the green fluorescence of GFP would be quenched, while the red fluorescence of mRFP would not [16]. In addition, the autophagosomes which contained mRFP-GFP-LC3 would be labeled with composite yellow fluorescent signal. The autophagosomes fusion with lysosomes is coincident with the fluorescent signal changing from yellow to red. Remarkably, only the yellow punctuate structure accumulated in the influenza-infected cells, indicating that autophagosomes seldom fused with lysosomes [6, 16]. Furthermore, after the inhibition of lysosomal proteolysis, the abundance of LC3-II markedly increases in the uninfected cells. On the contrary, in the cells infected with influenza A virus for 24 hours, accumulation of the LC3-II is reduced, suggesting that the influenza A virus infection restricts the degradation of autophagosomes by lysosomal proteolysis at late stages of infection [6].

It has been identified that M2 proteins of influenza A virus play a critical role in blocking autophagosome fusion with lysosome. Evidence showed that autophagosomes merely strongly accumulated in the M2-transfected A549 cells compared with the cells expressing other viral proteins of influenza A virus. In addition, the composite yellow fluorescent signal of GFP and mRFP was significantly increased, while red fluorescence was not, and the colocalization of GFP-LC3 and lysotracker staining was seldom observed, suggesting that autophagosomes accumulated without a concomitant with the increase of autolysosomes in the cells transfected with M2 plasmids [6, 16]. By contrast, in the cells infected with the viruses lacking M2, the accumulation of autophagosomes almost could not be detected, demonstrating that M2 is sufficient and essential to prevent autophagosome fusion with lysosome.

Interestingly, M2 localizes at cell membranes and perinuclear autophagic vesicles, which was identified by fluorescence microscopy. M2 even accumulated in some autophagosomes for the blockade of their fusion with lysosomes [6]. It is known that M2 mediates proton channel activity to acidify the interior of virions during the viral uncoating after fusing in endosome. However, suppressing this regulatory mechanism could not prevent the autophagosome accumulation, denying the hypothesis that M2 blocks autophagosome fusion with lysosome by regulating acidification of autophagosome membrane [31]. Another hypothesis is proposed that this function of M2 might be associated with Beclin-1 which is required for the fusion of autophagosomes with lysosomes. Indeed, the coimmunoprecipitation of M2 with Beclin-1 confirms this hypothesis, indicating that M2 may cause negative regulation of Beclin-1 to block membranes fusion of autophagosomes with lysosomes (Figure 1). However, the molecular basis and precise role of interaction between M2 and Beclin-1 still remain to be explored. Intriguingly, the C-terminal truncation of M2 protein containing only the first 60 amino acids still possesses the ability to upregulate the accumulation of autophagosomes and coimmunoprecipitate with Beclin-1, suggesting that the major functional domain of M2 to interact with Beclin-1 is in the first 60 N-terminal amino acids [6, 32, 33].

## 4. Autophagy Is Associated with the Accumulation of Viral RNA and Protein

To fully understand the interplay between the autophagy and viral production, it is usually critical to downregulate the activity of autophagy. The pharmacological inhibition and genetic manipulation technique are frequently employed in autophagy inhibition. Recent studies have suggested that the formation of phagophore membrane is stimulated by the class III phophatidylinositol 3-kinase (PI3K) complex, which is comprised of the PI3K, vacuolar protein sorting 34 (Vps34), p150, Atg14L, and Atg6 (Beclin-1 in mammalian cells) [10, 12, 25]. The activated Vps34/PI3K promotes the generation of PI3P, which is essential for the recruitment of binding additional function proteins. Recently, pharmacological methods are developed to use PI3K inhibitors for autophagic downregulation in vitro, including wortmannin, LY294002, and 3-MA [16]. It should be noted that the inhibition of the PI3K can cause marked decrease in viral titers and protein abundance at the early stage of influenza A virus infection. This effect vanished at the late stage of infection. Therefore, the PI3K inhibitor should not be employed for investigation of autophagy-related viral component accumulation during the initial infection [34]. The genetic manipulation technique was often used, such as knockout of essential Atg genes. Depletion of LC3 or Atg5 by specific RNA interference is also commonly used for autophagy inhibition [16, 35].

Autophagy is initially known as a catabolic process for the recycling of the cytoplasmic macromolecules; it may also serve as an anabolic pool for the replication and assembly of certain viruses, such as rotavirus and nidovirus, whose viral elements could colocalize with LC3 in the infected cells [36, 37]. Autophagy is also involved in the viral component accumulation of influenza A virus. Through pharmacological inhibition of autophagy, both the viral titers and tissue culture infectious dose (TCID_50_) of the influenza A virus (H9N2, H1N1) were significantly decreased in supernatants of infected A549 cells cultures [17, 38]. In contrast, the yield of virus was markedly reduced in the A549 cells by depletion of autophagy essential proteins. The similar experiment was performed in the MEF cells, but no significant changes of viral yield could be detected [6]. Moreover, the autophagy inhibition did not have a notable effect on the viral replication of H5N1 in vitro or in vivo [21]. These are likely due to the pathogenicity of influenza A virus and the peculiarities of host cells. Collectively, in the cells infected with low-pathogenic influenza A virus, autophagy may facilitate viral replication. Interference with autophagy releases more viral proteins from the autophagy-deficient cells compared with the autophagy competent cells. Recent studies reveal that the levels of viral proteins, including HA, M1, and M2, are increased in the autophagy competent cells [6, 17]. In general, the autophagy is involved in regulating viral antigen accumulation in the influenza-infected cells. It has been found that the autophagy-deficient cells release more viral antigens. Thus, the autophagy may contribute to reducing the antigenicity of influenza virus infection, and thereby the virus can avoid the host defense [23].

## 5. Autophagy Is Associated with Influenza A Virus-Induced Cell Death

The influenza A virus-induced cell death is associated with two major mechanisms: apoptosis induction and antiapoptosis autophagy suppression [39, 40]. It was reported that the influenza A virus-induced apoptotic cell deaths were significantly enhanced in the autophagy-deficient cells and the survival rate increased by knockout of M2 which has been known to block the autophagy completion. In general, autophagy can inhibit the apoptotic cell deaths caused by influenza A virus infection [6]. Recently, a novel protein of influenza A virus, polypeptide basic protein 1-frame2 (PB1-F2), has been identified as a second polypeptide which translates from the PB1 mRNA. PB1-F2 is an influenza A virus-encoded proapoptotic protein, which could make mitochondrial membranes unstable and permeable, thereby leading to apoptotic cell deaths [41, 42]. Therefore, influenza A virus encodes PB1-F2 to induce apoptosis and modulates M2 to promote apoptotic cell deaths indirectly by suppressing autophagy.

Although influenza A virus induces the apoptosis at the late stage of infection to accelerate the cell deaths, the virus limits apoptosis at the early stage for viral replication [22, 43]. Recent studies reveal that NS1 might be closely related to antiapoptosis among the proteins of influenza A virus, since, in the cells infected with delNS1 virus (H1N1 virus variant lacking the NS1 gene), the markers of apoptosis were presented earlier and more intensively in comparison with the control cells. This implies that the NS1 protein can delay the H1N1-induced apoptosis [20, 22]. It is observed that the NS1 interferes with apoptosis via targeting the protein kinase complex PI3K-AKT signaling pathway [22, 44]. The phosphorylated AKT only accumulated in the H1N1-infected cells but was seldom detected in delNS1-infected cells, indicating that the NS1 upregulated the activation of AKT that serves as an inducer of mTOR [45, 46]. However, NS1 has a negligible effect on accumulation of mTOR in the H1N1-infected cells [20]. Taken together, these data suggest that NS1 can upregulate the phosphorylation of AKT. Moreover, NS1 seems to be a positive regulator of autophagy, because generation of autophagosomes was decreased in the delNS1-infected cells [20, 47, 48].

Additionally, it has been reported that NS1 could bind to the double-stranded viral RNA-sensing protein kinase (PKR) and thereby inhibit the PKR-eIF2*α* signaling pathway[49]. Interestingly, this signaling pathway is critical for herpes simplex virus 1 (HSV-1) regulated autophagy. The viral protein Us11 of HSV-1 blocks autophagy by interaction with PKR [50, 51]. Therefore, it is possible that the NS1 protein of influenza A virus may regulate autophagy via interaction with the PKR-eIF2*α* signaling pathway. The full understanding of roles of the NS1 in the autophagy regulation requires further studies.

Thus far, the prosurvival mechanism of autophagy in apoptotic cell and its interaction with apoptosis are still unclear. However, the latest studies of the H5N1 virus indicate that the autophagic cell deaths may be another way that was utilized by the highly pathogenic influenza A virus to induce the cell death [40, 52]. Interference with autophagy by the pharmacological inhibitors could partially rescue the viability of H5N1-infected A549 cells, whereas the apoptosis inhibitor did not decrease the cell death rate under the same conditions. Consequently, the autophagic death is apparent to be the major cell death mechanism in the H5N1 infection [21, 28, 53]. Furthermore, evidence has showed that the viral titers of H5N1 and H1N1 were similar during the early infection, and then at later time points the H5N1 virus even replicated less efficiently than H1N1 did, ruling out the possibility that the higher mortality of the H5N1-infected cells is attributed to the more efficient replication [21].

It is thought that apoptotic bodies could reduce the immunogenicity of viral infection in comparison with necrotic cell debris [54]. Moreover, during the autophagy, the viral antigens were enwrapped in the membrane vesicles and escaped from the degradation for the transportation to immune systems of host cells via M2 [6]. The induction of apoptosis and the suppression of antiapoptosis autophagy both regulated by influenza A virus contribute to avoiding host defense, amplify virus replication, and finally trigger the death of host cells with little immunogenicity. The expression of PB1-F2 and the accumulation of autophagosomes are both presented at the terminal stage of infection, implying that the virus induces cell death without producing high immunogenicity [43].

## 6. Conclusion

Autophagy is initially known as a catabolic process for the recycling of the cytoplasmic macromolecules and also serves as a component of host defense. However, this mechanism is exploited by influenza A virus for its own benefits. On the one hand, the influenza A virus induces the formation of autophagosomes to accelerate replication, reduce antigenicity, evade host immune response, and trigger autophagic cell death. On the other hand, it blocks the maturation of autophagy to resist the degradation of autophagic contents by lysosomes, thereby leading to the accumulation of viral components and a positive regulation of apoptotic cell death. The in-depth analysis of molecular mechanisms underlying these processes will contribute to understanding of the specific autophagy pathways that are regulated by influenza A virus. Thus, the efficient countermeasures could be developed for the prevention of influenza virus infection.
